# DeepCubist: Molecular Generator for Designing Peptidomimetics based on Complex three-dimensional scaffolds

**DOI:** 10.1007/s10822-022-00493-y

**Published:** 2022-12-03

**Authors:** Kohei Umedera, Atsushi Yoshimori, Hengwei Chen, Hiroyuki Kouji, Hiroyuki Nakamura, Jürgen Bajorath

**Affiliations:** 1 School of Life Science and Technology, Tokyo Institute of Technology, 4259, Nagatsuta-cho, Midori-ku, 226-8503 Yokohama, Japan; 2 Department of Life Science Informatics, LIMES Program Unit Chemical Biology and Medicinal Chemistry, B-IT, Rheinische Friedrich-Wilhelms-Universität, Friedrich-Hirzebruch-Allee 5/6, D-53115 Bonn, Germany; 3 Institute for Theoretical Medicine, Inc, 26-1, Muraoka-Higashi 2-chome, 251-8555 Fujisawa, Kanagawa Japan; 4 Oita University Institute of Advanced Medicine, Inc, 17-20, Higashi Kasuga-machi, 870-0037 Oita City, Oita Japan; 5 Laboratory for Chemistry and Life Science, Institute of Innovative Research, Tokyo Institute of Technology, 4259, Nagatsuta-cho, Midori-ku, 226-8503 Yokohama, Japan

**Keywords:** 3D scaffold, Peptidomimetics, Deep learning, Generative modeling

## Abstract

Mimicking bioactive conformations of peptide segments involved in the formation of protein-protein interfaces with small molecules is thought to represent a promising strategy for the design of protein-protein interaction (PPI) inhibitors. For compound design, the use of three-dimensional (3D) scaffolds rich in sp3-centers makes it possible to precisely mimic bioactive peptide conformations. Herein, we introduce DeepCubist, a molecular generator for designing peptidomimetics based on 3D scaffolds. Firstly, enumerated 3D scaffolds are superposed on a target peptide conformation to identify a preferred template structure for designing peptidomimetics. Secondly, heteroatoms and unsaturated bonds are introduced into the template via a deep generative model to produce candidate compounds. DeepCubist was applied to design peptidomimetics of exemplary peptide turn, helix, and loop structures in pharmaceutical targets engaging in PPIs.

## Introduction

While small molecules have been essential sources for drugs targeting enzymes or G-protein-coupled receptors (GPCRs), lead compounds for specifically interfering with protein-protein interactions (PPIs) have been difficult to obtain using conventional small molecular design approaches [1]. Many contemporary compound collections are strongly enriched with aromatic and other unsaturated compounds, due to the preferential use of efficient synthetic approaches such as palladium coupling reactions, giving rise to planar compounds with limited or absent 3D features [2]. Especially for developing PPI inhibitors, such predominantly “flat” molecular templates are typically unsuitable. Hence, there is a growing interest in utilizing scaffolds for design that are rich in sp^3^ hybridized centers, thus having pronounced three-dimensional (3D) character and the ability to adopt compact molecular shapes.

Peptidomimetics are compounds designed to mimic the bioactive conformation of isolated peptides or specific peptide segments in proteins. While peptidomimetic design has a long tradition in drug discovery, it continues to be challenging, depending on the particular target and its ligand binding characteristics. This especially applies to the design of PPI inhibitors that are required to disrupt large protein-protein interfaces because in such cases, binding of a single small molecule must compensate for the free energy gained by large complementary protein surfaces forming many specific interactions.

Grossmann proposed four different classes of peptidomimetics: Class A-modified peptides (formed by α-amino acids with small backbone and side chain differences) ; Class B-modified peptides/foldamers (formed by amino acids with varying backbone and side chain iterations); Class C-structural mimetics (synthetic scaffolds with substitution sites corresponding to peptide side chain vectors); and Class D-mechanistic mimetics (small molecules mimicking the mode of action of peptides in the absence of side chain resemblance) [3]. Class C peptidomimetics completely replace the backbone of the parent peptide with a small molecule and reproduce the original side chain arrangements with its substituents. As a design premise, the development of peptidomimetics based on highly saturated bridged scaffolds, which can adopt complex shapes and enable the placement of functional groups in a variety of spatial arrangements for forming specific interactions can be expected to improve target selectivity and also potency compared to more planar scaffolds with less 3D character. This is the case because substitution sites in planar scaffolds have very limited ability to match the geometry of side chain arrangements across multiple amino acids in protein secondary structure elements. By contrast, 3D scaffolds increase the potential to precisely mimic side chains in peptides and match pharmacophores resulting from bioactive peptide conformations.

Scaffolds with new topology can in principle be obtained by computational enumeration of ring systems. For example, construction of a database called GDB4c containing 916,130 possible ring systems composed of up to four individual rings has been reported, enabling the discovery of new kinase inhibitors with previously unobserved chirality and shape [4]. For peptidomimetic design, 3D scaffolds must also be shape-diverse. Moreover, they must be capable of closely matching and replacing different peptide secondary structure motifs. At the same time, individual scaffolds should preferably be rigid to minimize entropic penalties upon binding.

Herein, we introduce DeepCubist, a molecular generator relying on deep learning for designing peptidomimetics based on previously unobserved 3D scaffolds and report initial proof-of-concept applications. In practice, the best fitting 3D scaffolds can be identified for turns, loops, or helical segments in structures of target proteins of interest and chemically diversified to obtain peptidomimetic candidates for interfering with PPIs. Hence, the DeepCubist approach complements and further extends structure-based design of PPI inhibitors by generating peptidomimetics with varying chemical features.

## Development of DeepCubist

### Methodological Concept

**Fig. 1:**
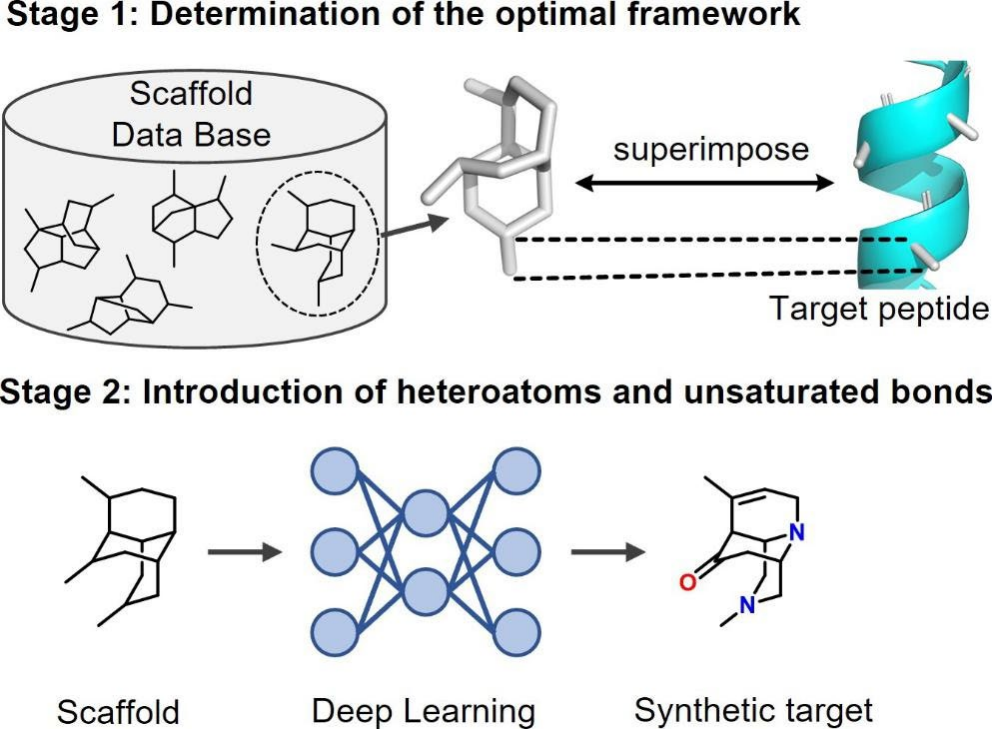
An overview of DeepCubist. The two major stages of the computational design approach are illustrated

DeepCubist is conceptualized to include two design stages, as illustrated in Fig. 1. At the first stage, a preferred scaffold for reproducing spatial side chain arrangements of a target peptide is determined. Therefore, a database of 3D scaffolds with methyl groups initially placed at three substituent positions is constructed, enabling initial superposition of the scaffold and Cα-Cβ bond of the target peptide. At the second stage, heteroatoms and unsaturated bonds are introduced into selected frameworks to provide further functionalities and support synthetic accessibility.

### Template scaffolds

**Fig. 2:**
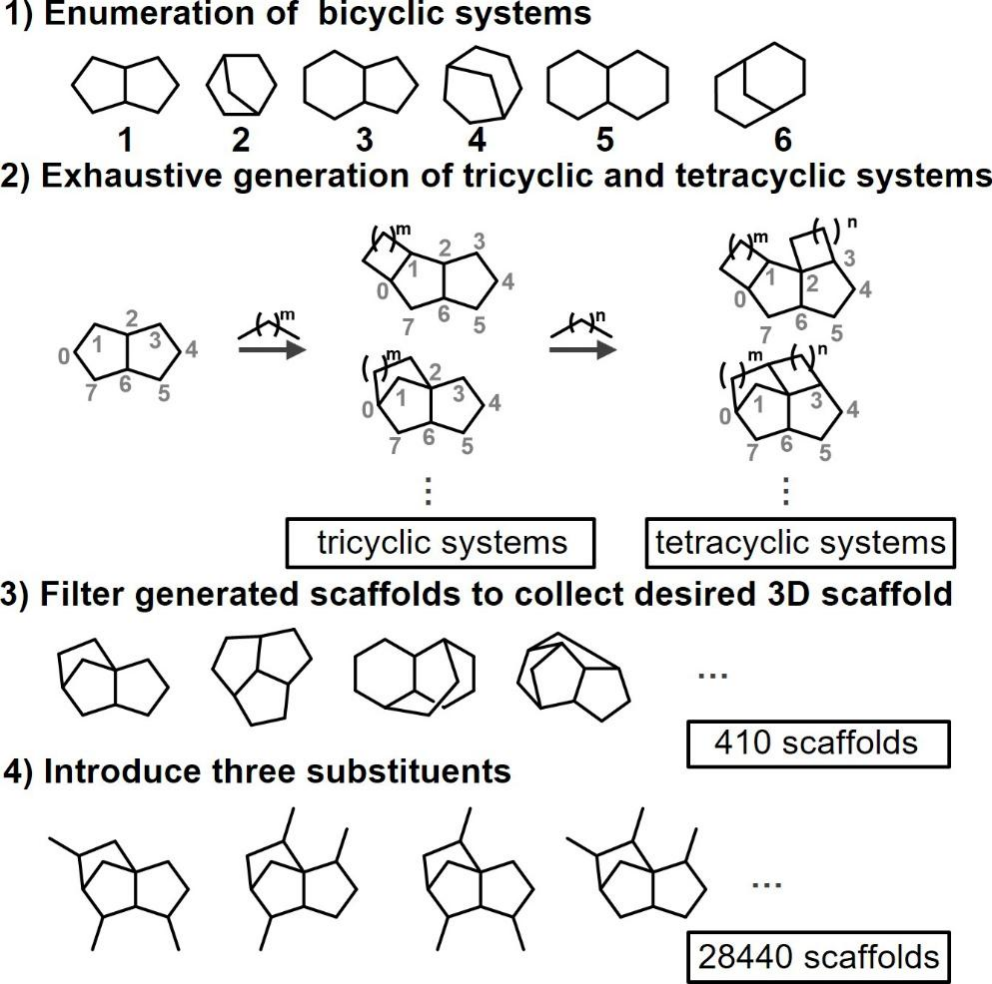
Generation of a 3D scaffold database

Construction of DeepCubist’s scaffold database began with defining a qualifying 3D scaffold as a *tricyclic or tetracyclic bridged ring system consisting of 5- and/or 6-membered rings*. This scaffold definition can be modified for different applications depending on the specific requirements. Our definition ensured that scaffold structures could be chemically diversified compared to, for example, bicyclic systems while restricting theoretically possible chemical complexity and hence increasing the likelihood of achieving synthetic accessibility. For our proof-of-concept investigation, so-defined scaffolds consisting of 10 to 14 carbon atoms were then systematically generated as illustrated in Fig. 2.

1) Six fused or bridged bicyclic systems consisting of 5- and/or 6-membered rings were computationally constructed as starting points (the number can be varied).

2) Tricyclic ring systems were then exhaustively generated by extensions of bicyclic systems with fragments comprising *m* carbon atoms added to any pair of ring atoms. From the resulting tricyclic ring systems, tetracyclic structures were obtained by addition of fragments with *n* carbon atoms to every atom pair of the tricyclic systems. Hence, (*m, n*) fragment combination were defined to obtain target scaffolds with 10 to 14 carbon atoms, depending on the size of the original bicyclic system.

For example, for bicyclic ring system **1** consisting of eight carbon atoms, (*m, n*) = {(2, 0), (1,1)} were used to exhaustively construct scaffolds with 10 carbons. As a result of these operations, a total of 1347 bridged ring systems were obtained at this stage.

3) The generated tri- and tetracyclic candidate structures were then filtered to collect chemically feasible 3D scaffolds with limited strain energy. Scaffold conformers were generated using the “ligand preparation” option of Discovery Studio 2020 [5] and conformers with a “clean energy” value of no more than 100 kcal/mol were collected, yielding 405 different 3D scaffolds with no chiral information.

4) Finally, combinations of three substituents were added to each 3D scaffold, in each case permitting the presence of at most one quaternary carbon for ease of synthesis (the number of substituents can vary). The introduction of substituent combinations resulted in a total of 28,440 unique carbon atom scaffolds with no chiral information. These carbon atom scaffolds can be classified as *3D cyclic skeletons*, following the hierarchical scaffold definition of Bemis & Murcko [6]. These skeletons served as input for the design of final 3D scaffolds containing heteroatoms and unsaturated bonds, as further described below.

### Generative model

Once 3D carbon skeletons are generated, they must be converted into chemically meaningful scaffolds. For this purpose, DeepCubist employs a deep generative model based on SMILES strings [7] as a standard text-based molecular representation. Such generative models have been applied, for example, to construct target-focused virtual libraries [8] or natural product-like compounds [9], demonstrating the ability to generate chemical structures of varying complexity. For training such models, SMILES of existing compounds are often augmented with randomized SMILES [10] to support learning of the chemical language encoded by string representations. As a deep learning architecture, a *transformer* model from natural language processing was selected [11]. Different from other sequence-to-sequence models, transformer models operate on the basis of *attention* mechanisms that identify and highly weight the most important representation elements for achieving accurate predictions during the training phase [11]. As further discussed below, the transformer model was trained to convert 3D carbon scaffolds into compounds containing heteroatoms and unsaturated bonds, that is, candidate compounds with chemical features amenable to synthesis.

### Source and target structures for training

**Fig. 3:**
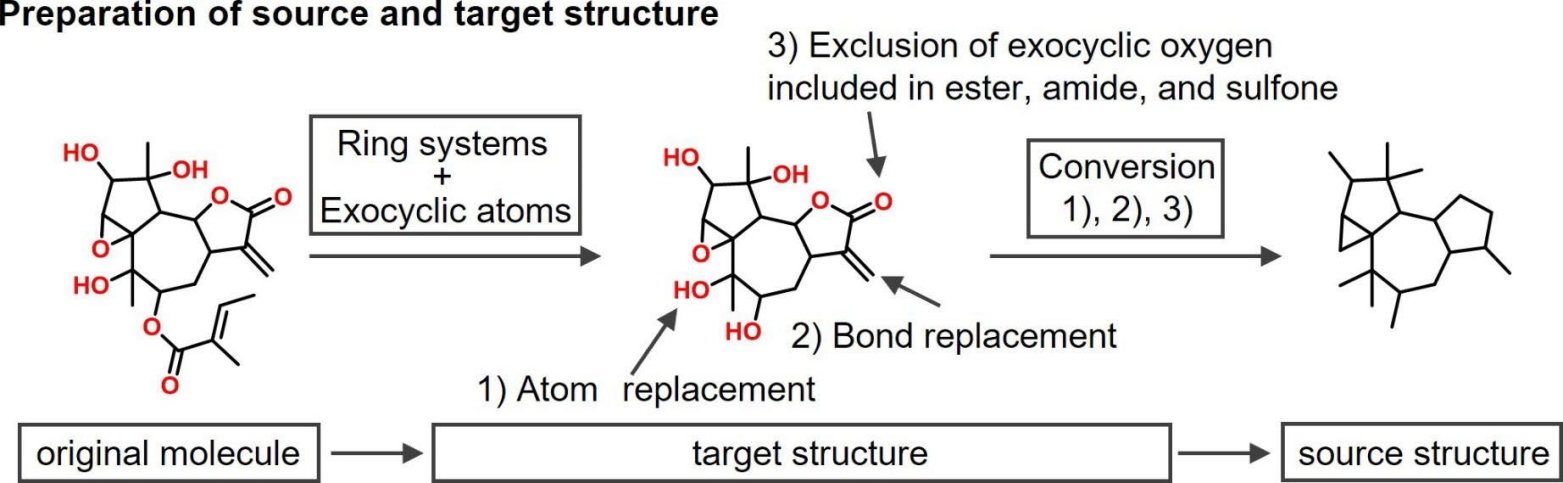
Source and target structures for training the transformer model

Drug- and natural product-like compounds were retrieved from ChEMBL version 30 [12] and COCONUT [13], a database of natural products, respectively. A total of 1,914,739 ChEMBL and 406,919 COCONUT compounds were obtained, referred to as original compounds. For model derivation, all possible *target* (output) structures were extracted from the original ChEMBL and COCONUT compounds by removing all exocyclic atoms from primary ring substituents and replacing removed fragments with a hydrogen atom (including, for example, ester, amide, or sulfone moieties), as illustrated in Fig. 3. Thus, target structures represented consistently defined scaffolds with primary substituents for deep learning and candidate structures for further chemical modifications. *Source* (input) structures were then obtained by converting target structures into cyclic skeletons through replacement of all heteroatoms with carbons and conversion of all bond orders to 1 (single bonds), as also illustrated in Fig. 3. After original compounds were decomposed, target structures with no more than eight atoms in individual rings and {C, N, O, S, F, Cl, Br, I} elements were collected for modeling. A total of 53,075 pairs of target and corresponding source structures were obtained. The use of these pairs of corresponding source and target structures for model derivation provided the basis for the generation of 3D scaffolds containing heteroatoms and unsaturated bonds from our newly generated database of 3D carbon skeletons described above. The 53,075 target structures were found to contain 268 of the total of 405 enumerated 3D scaffolds; hence, the remaining 137 scaffolds were novel.

### String representations for training

**Fig. 4:**
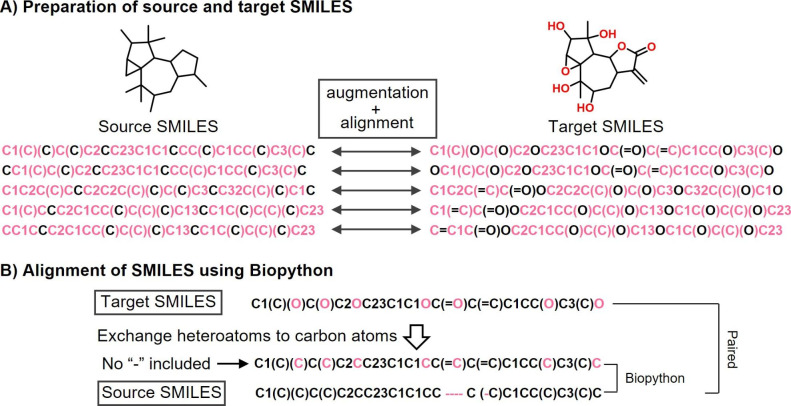
Molecular representations. (A) illustrates the generation and (B) the alignment of source and target SMILES for transformer training

For converting scaffolds into compounds using SMILES-based deep generative models, substitution sites in input structures are often marked as wild-card sites such as “*” to enable chemical diversification [14–16]. Furthermore, transformer-based retrosynthetic predictions have been improved by minimizing the edit distance between augmented input and output SMILES strings compared to unique canonical SMILES [17]. The edit distance between two SMILES strings is defined as the number of editing operations consisting of insertion, deletion, and substitution for transforming one string into the other. Corresponding SMILES representations with minimized edit distance closely link these representations for learning, which tends to reduce errors rates. In our study, this strategy was applied for model derivation, as illustrated in Fig. 4 A. After source and target SMILES were augmented by generating additional SMILES rooted at each atom using RDkit [18], newly generated SMILES with smallest edit distance were paired using the sequence alignment module implemented in Biopython [19], as shown in Fig. 4B. In accordance with the DeepCubist design strategy, heteroatoms in target SMILES were replaced with carbon atoms to obtain corresponding source SMILES. Then, the additional SMILES strings were aligned with the original source SMILES using the “pairwise2.align.globalxx” function of Biopython. In the alignment, identical characters obtain a score of 1, otherwise the score is 0. Since source structures were generated from target structures, gaps (“-”) in aligned SMILES strings can only occur in source SMILES.

### Model derivation

**Fig. 5:**
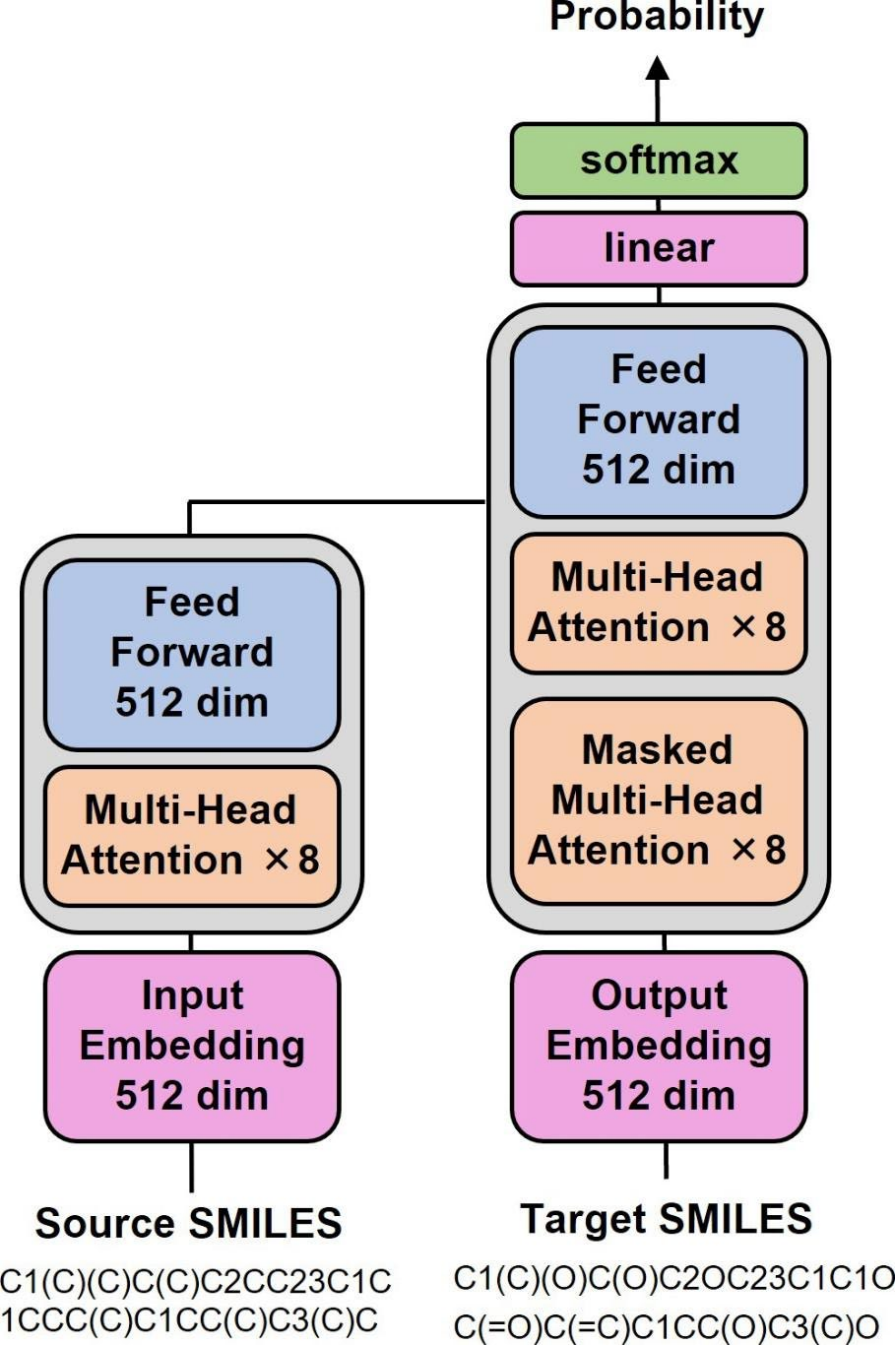
Transformer architecture and parameter settings

Pairs of source and target structures were randomly divided into 42,990 training (90%) and 4777 validation set (10%) instances. Following data separation, the SMILES augmentation and alignment steps were carried out. Original SMILES were iteratively augmented with randomized SMILES to obtain a total number of 168,137 pairs for training and 18,624 pairs for validation. A multi-head attention transformer model was constructed using Pytorch [20]. SMILES tokens were embedded in 512 dimensions, the number of heads was set as 8, the number of sub-layers in both encoder and decoder units was set to 3, and the dimensionality of the feed-forward network model was set to 512. For all remaining parameters, default settings were used. The model architecture including parameter settings is schematically illustrated in Fig. 5. For structure generation, SMILES tokens were sampled according to the learned probability distribution.

Scripts for the calculations and the data can be obtained via the following link:

https://www.dropbox.com/s/4gdhew9xjit43e4/DeepCubist_Materials.zip?dl=0.

## Exemplary applications

### Scaffold retention

**Fig. 6:**
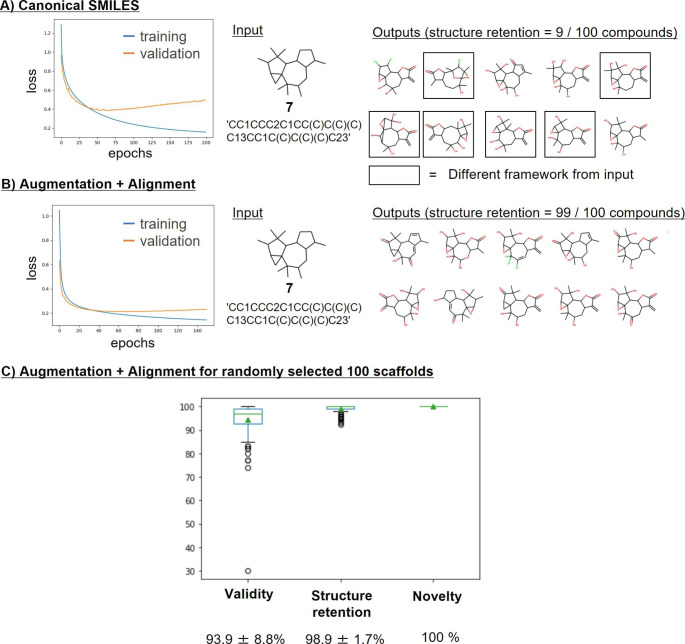
Model derivation. Results of transformer training and validation are shown including the evolution of the loss function (left) and input/output scaffolds (right) for (A) canonical or (B) augmented and aligned SMILES. (C) Analysis results are reported for 100 randomly selected skeletons

Initially, the effect of minimizing the edit distance between source and target SMILES was assessed. For transformer models trained using pairs of canonical SMILES (Fig. 6 A) or augmented and aligned SMILES (Fig. 6B), the loss value during validation was lower for augmented and aligned SMILES. Furthermore, for canonical SMILES, input structures were only poorly retained in target structures. After generating 100 unique structures using skeleton **7** as input with the model trained for 30 epochs, only nine target structures were found to completely retain the ring systems and the positions of substituents of source structures. In many cases, output structures with different ring sizes were obtained. By contrast, when the model was trained over 30 epochs with augmented and aligned SMILES, structure retention significantly increased. Using the same skeleton **7** as input, 99 of 100 newly generated structures exactly matched the composition and topology of the source structures.

The model was further evaluated using 100 randomly selected skeletons as input. In this case,  sampling of 100 new structures yielded ~ 94% string validity, ~ 99% input structure retention, and 100% output structure novelty (Fig. 6 C).

### Designing peptidomimetics

Having confirmed that the trained transformer model could effectively retain input structures during scaffold generation, DeepCubist was used to design exemplary peptidomimetics. To assess the general applicability of the approach, three types of peptide secondary structure were selected as starting points including peptide turns, helices, and loops. For each of the examples discussed below, a consistent number of 100 unique scaffolds was sampled. Furthermore, as an initial assessment of synthetic feasibility, the synthetic accessibility (SA) score according to Ertl & Schuffenhauer [21] were calculated using RDkit. The SA score ranges from 1 (easy synthesis) to 10 (very difficult) [21].

#### Turn mimetics

**Fig. 7:**
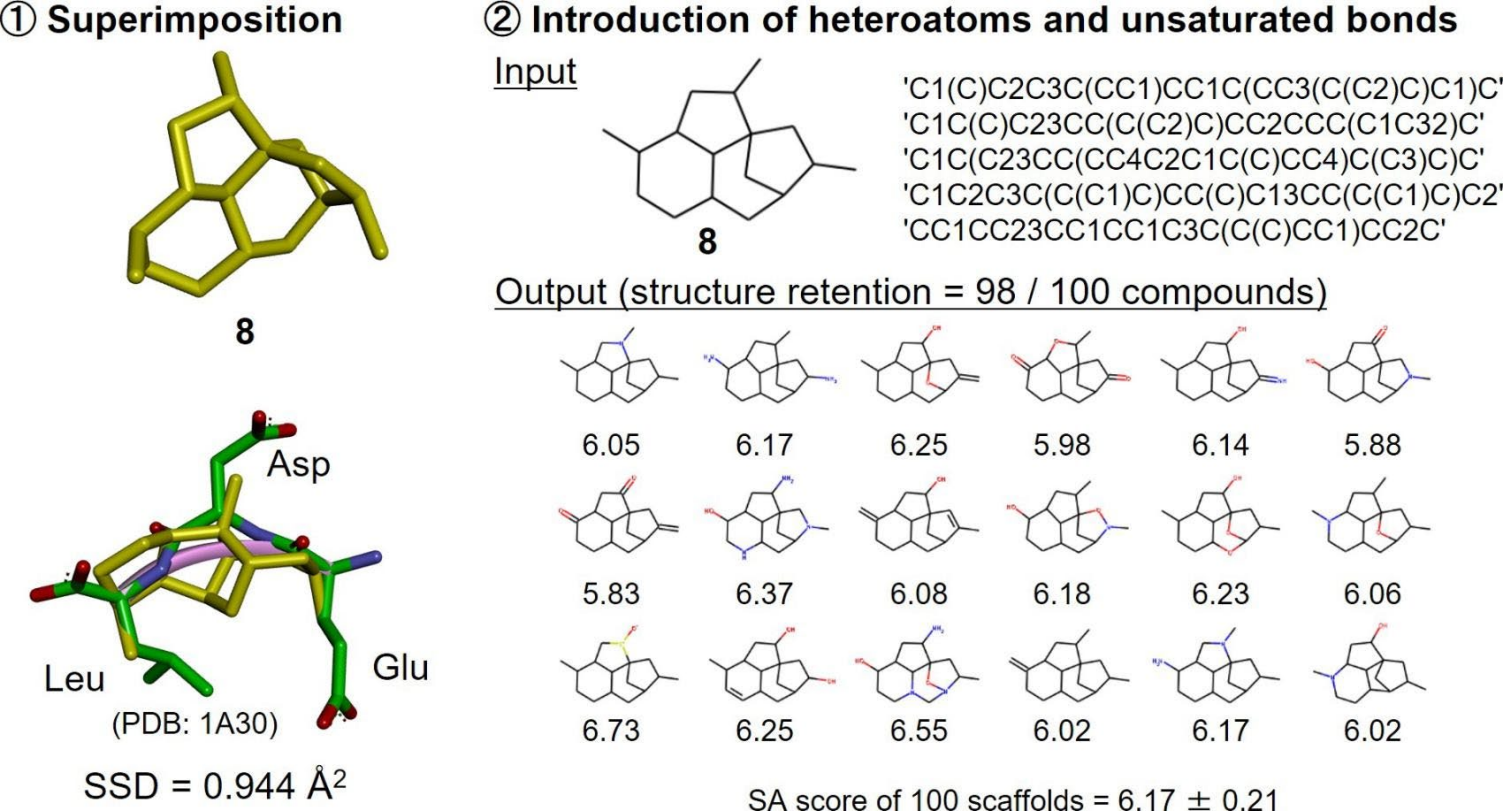
Design of turn mimetics. Shown is the skeleton that best reproduced the side chain orientations of the tripeptide turn in its bioactive conformation with the corresponding sum of squared deviations (SSD) value obtained after rigid-body superimposition of the corresponding atom pairs (including Cα and Cβ atoms of the peptide residues). In addition, exemplary output structures are shown. Figures 8 and 9 are represented accordingly

The tripeptide Glu-Asp-Leu is an inhibitor of HIV-1 protease. X-ray crystallography revealed that this tripeptide adopts a turn-like bioactive conformation in the active site of the enzyme [22]. The Cα-Cβ bonds of the tripeptide were superimposed on attachment points of 3D skeletons stored in DeepCubist’s database using the “rdAlignment.GetAlignmentTransform” module of RDkit. Tetracyclic skeleton **8** was discovered to best reproduce the side chain orientations of the tripeptide in its bioactive conformation with a sum of squared deviations (SSD) value of 0.944 Å^2^ (Fig. 7). The structure of skeleton **8** was not found in compounds used for model training. With five-fold augmentation of the skeleton **8** input SMILES, 100 unique output molecules retaining the input structure were obtained, 98 of which contained heteroatoms and double bonds. For the 100 structures, the mean SA score was 6.17, indicating reasonable synthetic accessibility.

#### Helix mimetics

**Fig. 8:**
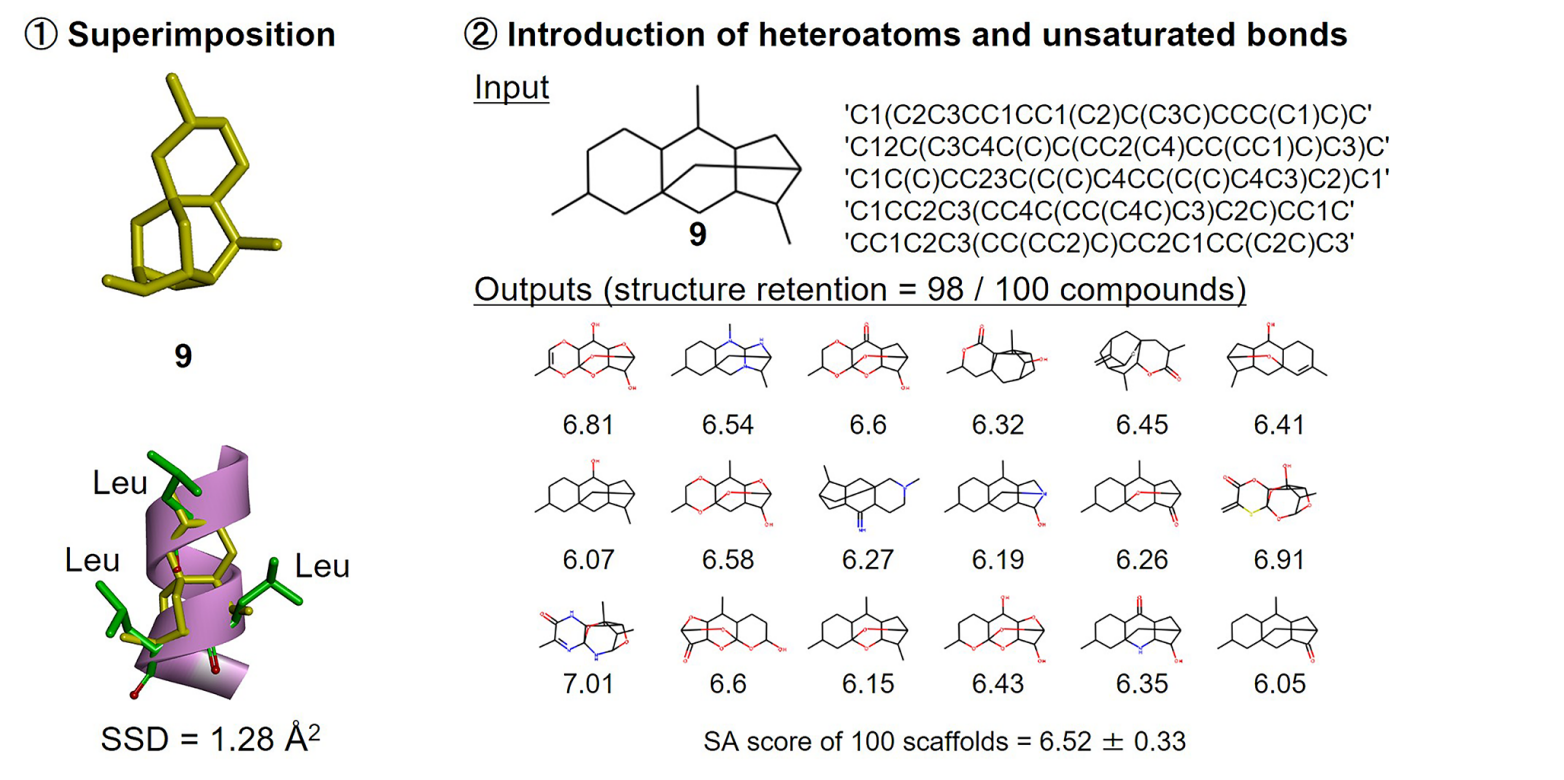
Design of helix mimetics

The peroxisome proliferator-activated receptor-γ (PPAR-γ) is a transcription factor that interacts with steroid receptor co-activating factor-1 (SRC-1) via the LxxLL motif presented by an α-helical peptide structure [23]. This LxxLL motif is widely observed at protein-protein interfaces [24]. If peptidomimetics were designed to precisely mimic varying conformations of this motif at different interfaces, selective PPI inhibitors might be obtained. Following the same calculation route as described above, DeepCubist identified tetracyclic skeleton **9** as the best available template for developing peptidomimetics, with an SSD value of 1.28 Å^2^ (Fig. 8). Of 100 newly generated 3D scaffolds, 98 retained the input structure. In this case, the mean SA score was 6.52.

#### Loop mimetics

**Fig. 9:**
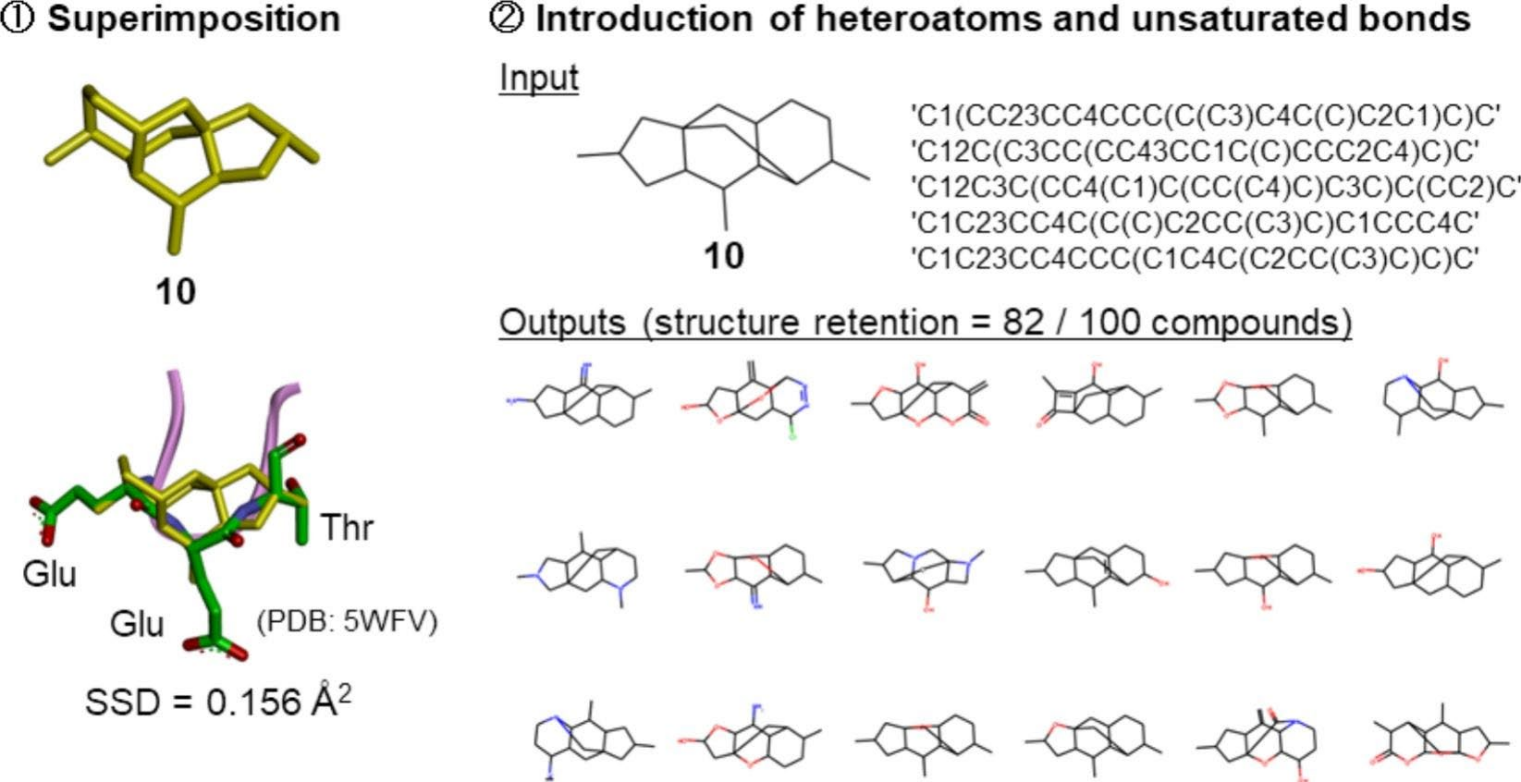
Design of loop mimetics

Modulation of nuclear factor erythroid 2-related factor(Nrf2) and Kelch-like-ECH-associated protein 1 (KEAP1) has been identified as an attractive therapeutic strategy for interfering with oxidative stress-related diseases such as cancer, neurodegenerative, cardiovascular, metabolic, or inflammatory diseases [25]. Nrf2 was identified to form a peptide loop structure (different from well-defined turns) at a hot spot of Nrf2-KEAP1 interaction, indicating the potential of loop mimetics as inhibitors of this interaction [26]. DeepCubist identified unique tetracyclic skeleton **10** hat –to our knowledge– has thus far not been considered in drug discovery and design (Fig. 9). The SSD value for the superimposition was 0.156 Å^2^. In this case, 85 of 100 unique output 3D scaffolds retained the input skeleton. The mean SA score for generated 100 scaffolds was 6.60.

## Conclusion

In this study, we have introduced DeepCubist, a molecular generator for the design of peptidomimetics. DeepCubist includes a specialized database of complex sp3-rich skeletons as templates for design and a transformer model trained to convert preferred skeletons into viable 3D scaffolds including heteroatoms and unsaturated bonds. Minimizing the edit distance of input and output SMILES was found to be a simple and effective way to control overfitting and tune the transformer for the construction of chemically meaningful 3D scaffolds retaining input structures. To establish proof-of-concept for DeepCubist’s design capacity, we have reported peptidomimetic designs for different peptide secondary structure motifs in high-profile therapeutic targets. While mostly favorable synthetic accessibility scores are obtained so far for newly generated 3D scaffolds, sp^3^-rich compounds have often more limited synthetic accessibility thancombinations of popular aromatic ring systems. Therefore, future work will primarily concentrate on ensuring a high degree of synthetic feasibility of newly generated scaffolds, for which different methodological avenues such as template- or reaction-based design can be considered.

## Data Availability

The analysis was exclusively carried out using publicly available data, as specified in the text. Code and the data sets used are made freely available.
